# Electroacupuncture Pretreatment Attenuates Intestinal Injury after Autogenous Orthotopic Liver Transplantation in Rats via the JAK/STAT Pathway

**DOI:** 10.1155/2020/9187406

**Published:** 2020-08-03

**Authors:** Lili Jia, Wenli Yu, Hongli Yu, Yiqi Weng

**Affiliations:** Department of Anesthesiology, Tianjin First Center Hospital, Tianjin 300192, China

## Abstract

**Background:**

Liver transplantation induces self-injury and affects remote organs, such as the lung, kidney, and intestine. Postoperative intestinal dysfunction has been associated with prolonged hospitalization and affects a patient's health and quality of life. Electroacupuncture (EA) has been proven effective in multiple organ protection. However, the potential mechanism underlying the protective effects of EA on intestinal injury after liver transplantation remains unclear.

**Methods:**

After establishing an autogenous orthotopic liver transplantation (AOLT) model, we studied the effects of EA pretreatment on intestinal injury after AOLT. We used the JAK2-specific inhibitor AG490 to explore the underlying mechanism. Histological analysis and apoptosis assays were used to evaluate intestinal injury. Oxidative stress index and inflammatory response were also measured after AOLT. Furthermore, we detected the phosphorylation levels of JAK2, STAT1, and STAT3 by Western blot.

**Results:**

We found that pretreatment with EA alleviated intestinal injury after AOLT, as shown by HE staining and TUNEL methods. EA pretreatment inhibited the expressions of p-JAK2, p-STAT1, and p-STAT3 in the intestines after AOLT. Upon treatment with JAK2-specific inhibitor AG490, intestinal injury was balanced.

**Conclusion:**

The data indicated EA pretreatment alleviated intestinal injury after AOLT by inhibiting the JAK/STAT signaling pathway. These results provide basic evidence to support the potential therapeutic efficacy of EA.

## 1. Introduction

Liver transplantation is the most effective method to address end-stage liver diseases. During the past few decades, remote organ injury has been widely studied during liver transplantation. Liver transplantation not only induced self-injury but also affected remote organs, such as the lung, kidney, and intestine [1–4]. With the development of surgical techniques, postoperative mortality and morbidity significantly reduced in recent years. However, postoperative intestinal dysfunction remains associated with prolonged hospitalization and affects a patient's health and quality of life. Therefore, numerous studies have been conducted on the prevention of intestinal injury, from clinical trials to animal studies. Based on Nozato et al.'s study [5], we established an autogenous orthotopic liver transplantation (AOLT) model in rats, which can well imitate the clinical surgery process. Although previous studies have explored various methods and drugs in ischemia organ injury, reported side effects have limited these studies from applying to clinical practice.

Acupuncture is a traditional Chinese medical treatment and is simple to perform, safe, and reliable. Electroacupuncture (EA) has been proven effective in multiple organ protection [6–8]. A previous study showed that excessive inflammation and oxidative stress response play vital roles in intestinal injury after liver ischemia-reperfusion [9]. Another study showed that EA protects against liver injury after liver ischemia-reperfusion [10]. Zusanli (ST36) is a widely used acupoint in humans to exert anti-inflammatory effects during acupuncture for treating pain or ischemia-reperfusion conditions [11, 12]. Herein, we used the ST36 acupoint to assess its protective effect against intestinal injury after AOLT in rats. Although EA has been increasingly used to treat various diseases, the mechanism remains unknown.

The Janus kinase/signal transducer and activator of the transcription (JAK/STAT) signaling pathway is involved in a wide range of distinct cellular processes, including inflammation, apoptosis, cell-cycle control, and development [13]. The JAK/STAT pathway comprises a family of receptor-associated cytosolic tyrosine kinases (JAKs) that phosphorylate tyrosine residues on bound transcription factors (STATs). JAK-mediated tyrosine phosphorylation of STAT family members enables the translocation of these transcription factors to the nucleus and leads to an augmentation of gene transcription [14, 15]. However, it is unclear whether the JAK/STAT signaling pathway is involved in EA pretreatment to prevent intestinal injury after AOLT. Based on the protective effect of EA on various tissues, our study is aimed at exploring the effect of EA on intestinal injury induced by AOLT in rats and at investigating the underlying mechanisms.

## 2. Methods and Materials

### 2.1. Animals

A total of 40 adult male Sprague-Dawley rats (220-250 g) were purchased from the People's Liberation Army Military Academy of Medical Sciences Laboratory Animal Center. The animals were housed in 12 h light-dark cycles with controlled room temperature and were fed with regular rat chow and water ad libitum but were fasted 12 h before experiments. Animals were randomly assigned into five groups: group A, sham operated (sham group); group B, autogenous orthotropic liver transplantation (AOLT group); group C, pretreated with EA (ST36, 1-2 mA, 2-100 Hz, 30 min) for 3 days+AOLT (EA+AOLT group); group D, pretreated with EA for 3 days+sham operated (EA+sham group); and group E, pretreated with EA for 3 days+AG490 (5 mg/kg, i.p., Selleck, USA) 30 min before establishing the AOLT model (EA+AOLT+AG490 group). The dosage was determined from a previous study [1]. The experimental procedures were conducted following the Guide for the Care and Use of Laboratory Animals and approved by the Institutional Animal Care and Use Committee.

### 2.2. Animal Model

**Figure 1:**
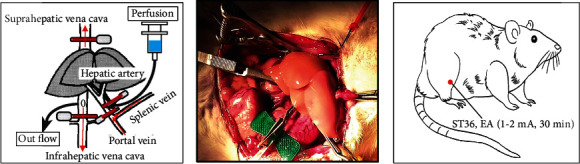
The autogenous orthotopic liver transplantation (AOLT) model in rats and electroacupuncture pretreatment on rats.

A rat AOLT model was established using a previously reported method [5]. Anesthesia was induced by inhalation of 3.0-4.0% isoflurane and maintained 1.5-2.0% isoflurane inhalation. During surgery, all rats were free to breathe O_2_ and were prone on a heating blanket. Rats in the sham group underwent laparotomy without performing AOLT as control. Total liver ischemia was induced by clamping the hepatic artery, the portal vein, suprahepatic vena cava (SHVC), and intrahepatic vena cava (IHVC). After clamping, the portal vein and IHVC were cannulated with a polyethylene tube, and the liver was perfused through the portal vein with 500 mL of heparinized cold saline (Jiangsu Wanbang Biochemical Pharmaceutical Co., Ltd., China) (2.5 IU/mL) to wash out all blood from the liver. The tubes were removed after perfusion, and the openings of tube insertion were closed. The SHVC and IHVC were then declamped immediately after repairing the vessels. After 45 min, the clamps were removed to allow the return of blood flow to the liver. After 6 h, the animals were euthanized by administering CO_2_ inhalation. Blood samples and intestinal tissue biopsies were taken. The tissues were fresh frozen for histological and biochemical evaluation. (Figure 1).

### 2.3. EA Pretreatment

Previous studies have shown that treatment with dilated 100 Hz and 2 Hz alternating frequency (2/100 Hz) EA stimulation of the ST36 acupoint can alleviate the inflammatory response caused by Complete Freund's adjuvant (CFA) [16, 17]. Zusanli acupoint is located 5 mm beneath the capitulum fibulae and lateral posterior to the knee joint. We performed EA pretreatment as described in a previous study [18]. Two acupuncture needles (diameter, 0.25 mm; length, 30 mm) were inserted into bilateral ST36 acupoints. Then, the two acupuncture needles were connected to the output of a Master-9 Pulse Stimulator (AMPI, Israel). EA parameters were set as follows: bidirectional symmetric square wave. The intensity was adjusted to induce moderate muscle contraction of the hind limb (1-2 mA), and the EA groups were subjected to EA with alternating frequencies and pulse waves. EA was applied 30 min/day from day 1 to day 3 (Figure 1).

### 2.4. Histological Examination

Formalin-fixed intestinal tissue was dehydrated, embedded in paraffin, and sliced into 4 *μ*m thick sections, which were stained with hematoxylin and eosin (HE). The damage of intestinal mucosal cells was analyzed by the Chiu score method [19]: 0—normal mucosa, 1—the tip of the hair on the top of the subcutaneous gap increases, 2—moderate separation of the epithelium and lamina, 3—when there are a large number of villi on both sides of the top of the villi with partial villi, 4—villous damage with a large number of inherent layer of capillary exposed, and 5—inherent layer damage of bleeding and ulcers.

### 2.5. Apoptosis Assay

To detect the apoptotic positive cells, terminal deoxynucleotidyl transferase-mediated digoxigenin-labeled UTP nick end labeling assay (TUNEL) was performed using an ApopTag peroxidase in situ cell death detection kit (Roche, Basal, Switzerland). Briefly, the 4 *μ*m thick paraffin sections were deparaffinized, treated with 0.1% TritonX-100, and incubated at 37°C for 8 min in a humidified atmosphere. PBS wash was performed 3 times. Then, the sections were further incubated with a mixture of marker and enzyme solution at 37°C for 1 h. PBS wash was performed 3 times, and the samples were observed under a fluorescence microscope (Olympus, Japan) at 450-550 nm. Five high-power fields were randomly selected to determine the percentage of apoptotic cells.

### 2.6. Enzyme-Linked Immunosorbent Assay (ELISA)

Blood samples were collected from all rats. The serum levels of tumor necrosis factor-*α* (TNF-*α*), high-mobility group box-1 (HMGB-1), intestinal fatty acid-binding protein (IFABP), and endotoxin (LPS) were examined using an ELISA kit (Shanghai Baiwo Co., Ltd., China) according to the manufacturer's instructions [20].

### 2.7. Wet/Dry (*W*/*D*) Ratio of the Intestine

To test the edema of intestinal after AOLT 6 h following reperfusion, 5 cm of ileum tissue at 2 cm from the back of the blind was harvested, cleaned by removing blood and water, and weighed. Then, the sample was incubated at 70°C for 24 h and weighed. The *W*/*D* ratio was calculated as the ratio of the wet weight to the dry weight of the intestine.

### 2.8. Oxidative Stress

The levels of malondialdehyde (MDA) and superoxide dismutase (SOD) were determined using assay kits (Nanjing Institute Co., Ltd.) according to the manufacturer's instructions [20]. The tissue MDA level was determined by the Esterbauer and Cheeseman method based on its reaction with thiobarbituric acid at 90-100°C; absorbance was measured at 532 nm. MDA reacts with thiobarbituric acid (TBA) and produces a pink pigment, which has a maximum absorption at 532 nm. The value of each sample was obtained from the standard curve and was expressed as *μ*mol/g tissue. SOD activity was measured according to the Paoletti and Mocali method. In this assay, superoxide anion was generated from molecular oxygen in the presence of EDTA, manganese II chloride, and mercaptoethanol. Nicotinamide adenine dinucleotide phosphate oxidation was linked to the availability of superoxide anions in the medium.

### 2.9. Immunohistochemistry

Immunohistochemical staining of the intestinal tissue was performed on formalin-fixed paraffin sections using a microwave-based technique. The 4 *μ*m thick sections of the fixed intestines were dewaxed with xylene, hydrated in graded concentrations of ethanol, and treated with 0.3% hydrogen peroxide for 10 min to quench endogenous peroxidase. The sections were heated in a microwave oven in sodium citrate (pH = 6.0) at 95-99°C for 15 min and then cooled at room temperature. The sections were incubated in 5% blocking serum for 30 min and then in primary antibodies (Cleaved Caspase-3: 1 : 1 000, from Cell Signaling Technology, Beverly, MA, USA) at 4°C overnight. They were subsequently incubated with biotinylated secondary antibodies for 30 min and finally counterstained with hematoxylin.

### 2.10. Western Blot

The protein lysates were prepared from frozen tissues in ice-cold RIPA buffer (Sigma-Aldrich). The extracted protein was separated in a 10% sodium dodecyl sulfate- (SDS-) PAGE and then electrophoretically transferred to a nitrocellulose membrane (Hybond, Amersham Biosciences, Little Chalfont, UK). Membranes were blocked with 5% nonfat milk powder in TBS for 1 h at room temperature. These membranes were subjected to immunoblot analysis with antibodies to JAK2, p-JAK2, STAT1, p-STAT1, STAT3, and p-STAT3. The protein antibody immune complexes were detected with horseradish peroxidase-conjugated secondary antibodies and enhanced chemiluminescence reagents (Pierce Biotechnology, Rockford, IL). GAPDH was used as an internal control to calculate the ratio of optical density, and values were compared with those of sham controls.

### 2.11. Statistical Analysis

All values are given as the mean ± SD. Statistical analysis was carried out using GraphPad Prism 8.0 software (GraphPad Software, USA). One-way analysis of variance was used to compare the measurement data between groups. *P* < 0.05 was considered significant.

## 3. Results

### 3.1. EA Pretreatment Significantly Decreases Intestinal Histology Injury

**Figure 2:**
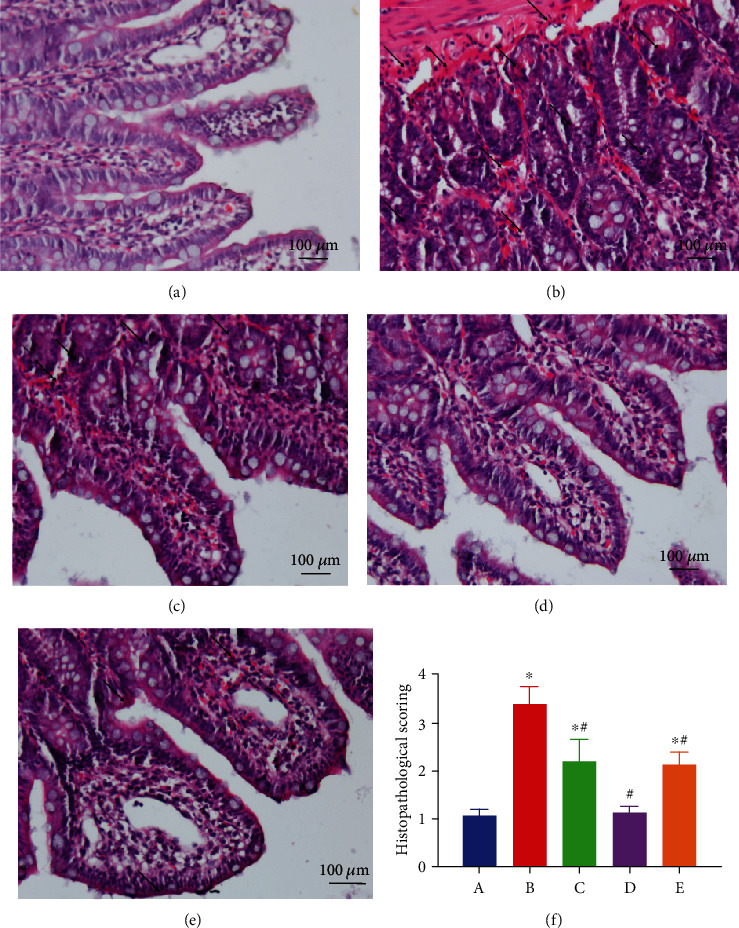
EA pretreatment significantly decreases intestinal histology injury (magnification, ×200). Representative microphotographs were taken from the intestine of the sham (a), AOLT (b), EA+AOLT (c), EA+sham (d), and EA+AOLT+AG490 (e) groups at the time point of 6 h after AOLT in rats. (f) Histopathological scoring was calculated in each group: (A–E) the sham, AOLT, EA+AOLT, EA+sham, and EA+AOLT+AG490 groups. Data were represented as the mean ± SD (*n* = 8, per group). ^∗^*P* < 0.05 vs. the sham group. ^#^*P* < 0.05 vs. the AOLT group.

To examine the protective effect of EA pretreatment against intestinal injury after AOLT, each rat was analyzed by histopathology, and the intestinal mucous damage score was calculated. A normal histological structure was observed in the sham group by HE staining. Severe disruption of structural integrity in brush border, including loss of mucus, villi, and widespread necrotic area, was observed in the AOLT group. However, these damages were improved in the EA pretreatment group. The intestinal mucous damage score significantly increased in the AOLT group compared with the sham group. EA pretreatment combined with AG490 improved intestinal damage; no significant differences were observed between the EA+sham group and the EA+AOLT+AG490 group (Figures 2).

### 3.2. EA Pretreatment Alleviates *W*/*D* Ratio of the Intestine

**Figure 3:**
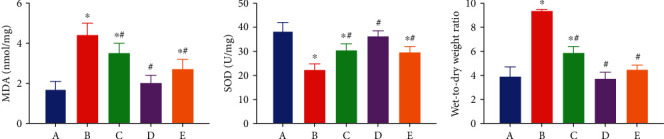
Effects of EA on the plasma level of MDA, SOD, and the *W*/*D* ratio following AOLT-induced intestinal injury. The levels of plasma MDA, SOD, and the *W*/*D* ratio were measured at 6 h following AOLT. (A–E) The sham, AOLT, EA+AOLT, EA+sham, and EA+AOLT+AG490 groups. Date were represented as the mean ± SD (*n* = 8, per group). ^∗^*P* < 0.05 vs. the sham group. ^#^*P* < 0.05 vs. the AOLT group.

To test intestinal edema after AOLT, we calculated the *W*/*D* ratio changes in all groups. As shown in Table 1, EA pretreatment significantly reduced the *W*/*D* ratio caused by AOLT. When compared with the AOLT group, the intestinal *W*/*D* ratio decreased in the EA+sham and EA+AOLT+AG490 groups. The EA+sham group did not show a significant difference from the sham group (Table 1, Figure 3).

### 3.3. EA Pretreatment Decreases Intestinal Inflammatory Reaction and Oxidative Stress

**Figure 4:**
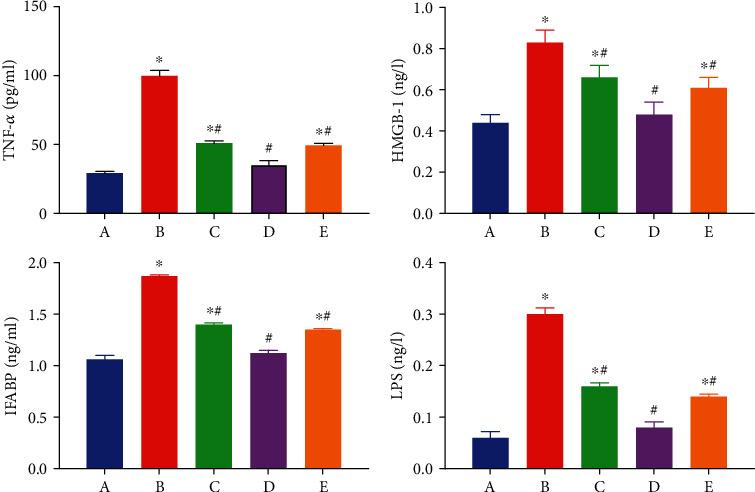
Effects of EA on the plasma level of TNF-*α*, HMGB-1, IFABP, and LPS following AOLT induced intestinal injury. The levels of plasma TNF-*α*, HMGB-1, IFABP, and LPS were measured at 6 h following AOLT. (A–E) The sham, AOLT, EA+AOLT, EA+sham, and EA+AOLT+AG490 groups. Date were represented as the mean ± SD (*n* = 8, per group). ^∗^*P* < 0.05 vs. sham group. ^#^*P* < 0.05 vs. the AOLT group.

As aforementioned, EA application was shown to protect against intestinal injury following AOLT. However, the underlying mechanisms remain unknown. Severe intestinal damage was found to be accompanied by oxidative stress and inflammatory reaction activation. The levels of TNF-*α*, HMGB-1, iFABP, LPS, and MDA increased markedly in the AOLT group. However, the application of EA effectively balanced oxidative stress and inflammatory reactions. The levels of TNF-*α*, HMGB-1, iFABP, LPS, and MDA decreased, whereas the level of SOD increased in EA+AOLT group. In the EA+sham group, EA had no effect on oxidative stress and inflammatory response, which were comparable with the results observed in the sham group. Results from the EA+AOLT+AG490 group were also similar to those from the EA+sham group (Figures 4 and 3).

### 3.4. EA Pretreatment Inhibits Intestinal P-JAK2, P-STAT1, and P-STAT3 Protein Expressions

**Figure 5:**
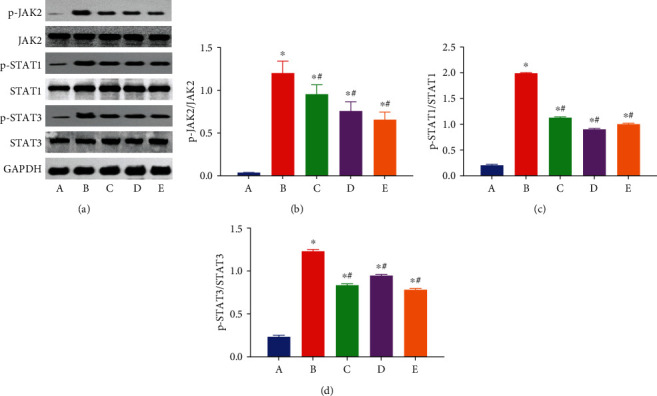
EA inhibited the phosphorylations of JAK2, STAT1, and STAT3. Representative Western blots for the phosphorylations of JAK2, STAT1, and STAT3 (a) of the intestine were detected after 6 h of AOLT; (A–E) The sham, AOLT, EA+AOLT, EA+sham, and EA+AOLT+AG490 groups. Densitometry analysis of Western blots for the ratio of p-JAK2/JAK2 (b), p-STAT1/STAT1 (c), and p-STAT3/STAT3 (d). Data were represented as the mean ± SD (*n* = 8, per group). ^∗^*P* < 0.05 vs. the sham group. ^#^*P* < 0.05 vs. the AOLT group.

The JAK/STAT pathway plays a vital role in regulating the immune response. Western blot analysis was performed to demonstrate the effect of EA on JAK/STAT pathway proteins. JAK2, STAT1, and STAT3 proteins were mainly expressed in intestinal cells. The expressions of p-JAK2, p-STAT1, and p-STAT3 proteins significantly increased in intestines subjected to AOLT (*P* < 0.05 vs. the sham group). However, EA application decreased the expression of p-JAK2, p-STAT1, and p-STAT3 proteins (*P* < 0.05 vs. the AOLT group). Treatment with AG490 reduced the expressions of p-JAK2, p-STAT1, and p-STAT3 proteins (*P* < 0.05 vs. the AOLT group). These results demonstrated that EA inhibited the JAK/STAT signaling pathway in intestinal injury after AOLT. The JAK/STAT signaling pathway was active in the AOLT group (Figure 5).

### 3.5. EA Pretreatment Decreases the Expression of Cleaved Caspase-3 in the Intestine

**Figure 6:**
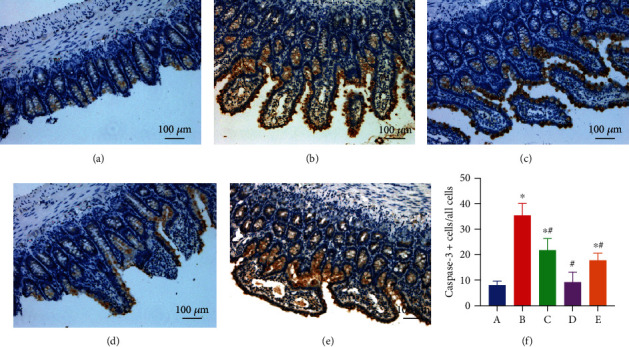
The effect of EA on AOLT induced Cleaved Caspase-3 of intestinal cells (magnification, ×200). Representative microphotographs were taken from the intestine of the sham (a), AOLT (b), EA+AOLT (c), EA+sham (d), and EA+AOLT+AG490 (e) groups at the time point of 6 h after AOLT. (f) Quantification of Cleaved Caspase-3 positive cells were counted in each group: (A–E) the sham, AOLT, EA+AOLT, EA+sham, and EA+AOLT+AG490 groups.

From the immunohistochemical staining, intense staining for active Cleaved Caspase-3 in the cytoplasm of epithelial cells of rats subjected to AOLT was observed. However, very few Cleaved Caspase-3 immunoreactive cells were observed in the sham and EA+sham groups. These results again showed the tissue-protective effect of EA against intestinal injury after AOLT (Figure 6). When pretreated with EA and AG490, the EA+sham and EA+AOLT+AG490 groups did not show a significant difference from the sham group.

### 3.6. EA Pretreatment Attenuates Intestinal Epithelial Cell Apoptosis

**Figure 7:**
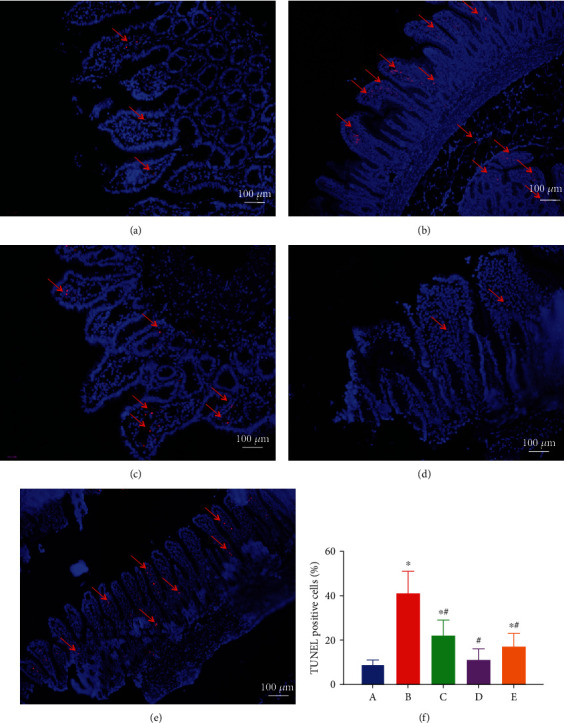
The effect of EA on AOLT induced apoptosis of intestinal cells (magnification, ×200). Representative microphotographs were taken from the intestine of the sham (a), AOLT (b), EA+AOLT (c), EA+sham (d), and EA+AOLT+AG490 (e) groups at the time point of 6 h after AOLT. Apoptosis was evaluated by TUNEL staining. Quantification of TUNEL-positive cells was counted following AOLT (f). ^∗^*P* < 0.05 vs. the sham group. ^#^*P* < 0.05 vs. the AOLT group.

TUNEL assay was used to evaluate the apoptosis of tubular epithelial cells induced by AOLT. A large number of apoptotic epithelial cells were visible in the intestines subjected to AOLT (*P* < 0.05 vs. the sham group). EA application was associated with the occurrence of apoptosis of epithelial cells, which was less than that observed with the AOLT group (*P* < 0.05). In the EA+sham group, EA treatment had no effect on apoptotic epithelial cells, comparable with the sham group. The EA+AOLT+AG490 group had similar results as the sham group (Figure 7).

## 4. Discussion

**Figure 8:**
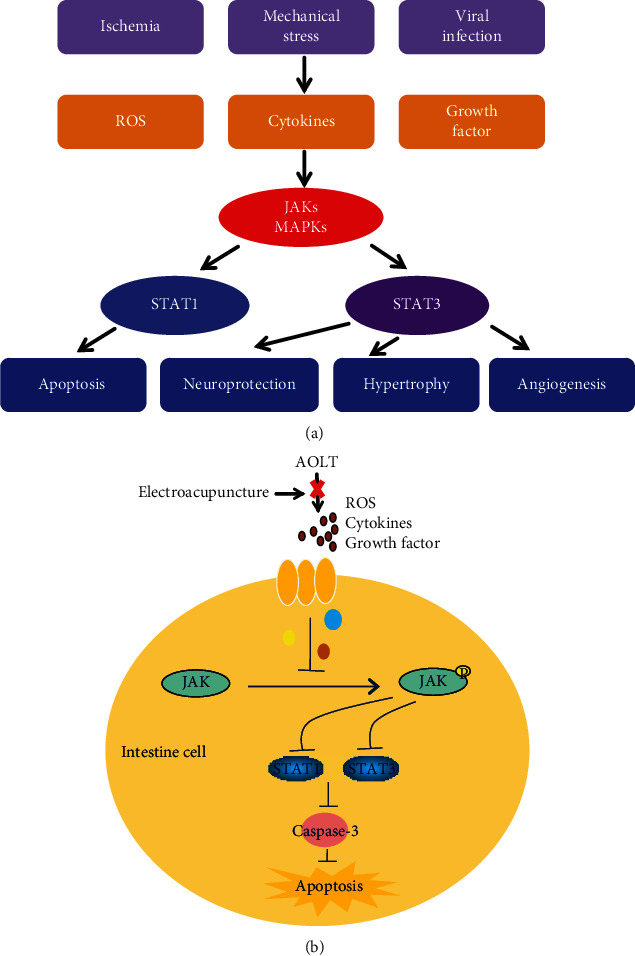
The possible mechanism of EA on AOLT induced apoptosis of intestine (a). The possible mechanism involved in the JAK/STAT pathway (b). EA pretreatment may have protective effects on AOLT-induced intestine injury by inhibiting JAK/STAT pathway.

We found that AOLT-induced intestinal injury in rats. We determined whether EA affected the results. Pretreatment with EA alleviated intestinal injury after AOLT, as shown by HE staining and TUNEL methods. Compared with the sham group, the EA+sham group did not produce adverse reactions as normal rats. We explored the potential mechanisms underlying EA pretreatment. EA pretreatment inhibited the expressions of p-JAK2, p-STAT1, and p-STAT3 in intestines after AOLT. When treated with JAK2-specific inhibitor AG490, intestinal injury was balanced. This indicated that EA pretreatment alleviated intestinal injury after AOLT by inhibiting the JAK/STAT signaling pathway and provides basic evidence supporting its potential therapeutic efficacy (Figure 8).

AOLT blocks the return of venous blood in the lower extremities, which induces a systemic response and a release of harmful substances that may damage remote organs, including the intestines [21–24]. After AOLT in rats, blood flow is blocked, and intestinal injury undergoes two processes: ischemia and secondary reperfusion injury [20]. Due to intestinal hyperemia and congestion during liver transplantation, intestinal peristalsis and barriers are often impaired. Intestinal erogenous endotoxin and bacteria enter the blood or lymphatic system and are transferred to other organs, which may lead to multiple organs dysfunction and systemic inflammatory response [25]. Intestinal injury is a complex, multifactorial, and pathophysiological process that involves dysfunction of bacterial translocation, absorption, and production of reactive oxygen species, cytokines, nitric oxide, and initiates multiorgan dysfunction syndrome [4, 26]. A recent study found that intestinal barrier destruction was widely observed during liver transplantation [27]. Other studies have also explored the possible mechanism of intestinal injury caused by liver transplantation. TLR4/NF-*κ*B signaling pathway activation-induced cell apoptosis was involved in intestinal injury during liver transplantation [28]. In our study, we found that the intestinal injury score increased after AOLT. Our previous study showed that AOLT induced remote organ injury after 6 h reperfusion, and the inflammatory response was obvious [29]. Therefore, in the present study, we selected 6 h after reperfusion to measure the intestinal injuries caused by AOLT.

Numerous strategies have been designed to reduce intestinal injury after AOLT [30]. Although some drugs have been successfully used to reduce intestinal injury in animal models, few can be used in clinical settings or may not be available during operations. Acupuncture has been used to control weight [31], reduce epilepsy [32], improve learning and memory disorders [33], and reduce pain [34–37]. EA is a complementary alternative medicine approach and involves applying an electrical current to acupuncture points. Zusanli is an important acupuncture point commonly used in acupuncture practices to promote blood circulation and can also cure peripheral soft tissue inflammation and ischemia-reperfusion [6, 38–41]. Indeed, several studies have demonstrated that EA significantly attenuated apoptosis in the brain, heart, and intestinal ischemia-reperfusion [42–44]. In our study, inflammatory reaction markers (TNF-*α*, HMGB-1) and oxidative stress factors (MDA) increased significantly in the AOLT group. However, in the EA+AOLT group, apoptosis of epithelial cells resulting from the intestinal injury was significantly decreased. Caspase-3, which participates in the apoptosis pathway in the intestinal mucosa, was also markedly reduced in the EA+AOLT group, which confirmed the decreased disruption of the structural integrity of intestinal mucosa, as observed by HE staining. This study evaluated the protective and antiapoptotic effects of EA on intestinal architecture and apoptosis in intestinal injury.

JAK/STAT is involved in many pathophysiological processes in the body [45, 46]. JAK2 is a member of the Janus kinase family and is involved in immune, hematopoietic, neural, and other signal transduction systems [47]. A recent study showed that EA has a protective effect on focal cerebral ischemia by inhibiting JAK2 phosphorylated activation in rats [48]. AG490 is a specific inhibitor of JAK2 which can inhibit the activation of JAK2 and downregulate the phosphorylation of STATs. The JAK/STAT signaling pathway is involved in ischemia-reperfusion processes. Our previous study showed that the JAK/STAT signaling pathway was inhibited by propofol in the hippocampus [29]. To confirm the hypothesis that the JAK/STAT signaling pathway participated in regulating apoptotic process in our model, AG490 was given in the EA+AOLT group. Our study showed that EA+AG490 significantly improved intestinal apoptosis and reduced the expression of Cleaved Caspase-3 protein following AOLT. Our data also showed significantly decreased expressions of p-JAK2 in the EA and EA+AG490 groups, accompanied by the downregulation of STAT1 and STAT3 phosphorylation. Our results indicated that EA significantly protected intestinal injury after AOLT in rats by inhibiting the activation of the JAK/STAT signaling pathway.

Taken together, intestinal injury contributed to the damage of intestinal function and histological structure and enhanced the apoptosis of intestinal epithelial cells and the expression of protein Cleaved Caspase-3. We demonstrated that EA pretreatment attenuated intestinal injury by inhibiting the JAK/STAT signaling pathway in a rat AOLT model. Zusanli is a classic and preferred acupoint for treating gastrointestinal diseases and is widely used in clinical practice. Its therapeutic effect is recognized by many medical workers and patients. EA is a branch of acupuncture and moxibustion therapy. Compared with classic acupuncture, it has many advantages, such as good therapeutic effects, a wide range, ease of stimulation control, and continuous needle movements [49]. However, there are several limitations to our study. This study was based on our previous study; we could have used gene sequencing technology to explore the involved mechanism. Then, we would explore the mechanism in vivo and investigate whether this mechanism functioned at the cellular level. Finally, we need to apply this technique in clinical practice. To make progress on this front, our research team has begun a single-center study on the protective effect of EA pretreatment on postoperative organs.

In conclusion, our study showed that EA reduced intestinal injury against AOLT, at least in part through its inhibitory effects on the injury-induced activation of the JAK/STAT signaling pathway. If extrapolated to a clinical setting, EA has the potential to serve as a clinical strategy for preventing perioperative intestinal injury after AOLT.

## Figures and Tables

**Table 1:** *W*/*D* ratio of experiment rats (*n* = 8, $\bar{x}\pm s$).

Groups	*W*/*D* ratio
Sham	3.89 ± 0.81
AOLT	9.35 ± 0.13^∗^
EA+AOLT	5.85 ± 0.55^∗^^#^
EA+sham	3.70 ± 0.57^#^
EA+AOLT+AG490	4.45 ± 0.41^#^

Data presented are the mean ± SD. ^∗^*P* < 0.05 vs. the sham group. ^#^*P* < 0.05 vs. the AOLT group.

## Data Availability

The data used to support the findings of this study are available from the corresponding author upon request.
